# PACE - The first placebo controlled trial of paracetamol for acute low back pain: design of a randomised controlled trial

**DOI:** 10.1186/1471-2474-11-169

**Published:** 2010-07-23

**Authors:** Christopher M Williams, Jane Latimer, Christopher G Maher, Andrew J McLachlan, Chris W Cooper, Mark J Hancock, Richard O Day, James H McAuley, Chung-Wei Christine Lin

**Affiliations:** 1 The George Institute for International Health, University of Sydney, PO Box M201 Missenden Rd, Camperdown, NSW 2040, Australia; 2 Back Pain Research Group, Faculty of Health Sciences, University of Sydney, PO Box 170, Lidcombe, NSW 1825, Australia; 3 Faculty of Pharmacy and Centre for Education and Research in Ageing, University of Sydney, Sydney, NSW 2006, Australia; 4 Clinical Pharmacology UNSW and St Vincent's Hospital, Darlinghurst, NSW, Australia, 2010; 5 Discipline of General Practice, Sydney Medical School, Balmain Hospital, 37A Booth Street, Balmain, NSW 2041, Australia

## Abstract

**Background:**

Clinical practice guidelines recommend that the initial treatment of acute low back pain (LBP) should consist of advice to stay active and regular simple analgesics such as paracetamol 4 g daily. Despite this recommendation in all international LBP guidelines there are no placebo controlled trials assessing the efficacy of paracetamol for LBP at any dose or dose regimen. This study aims to determine whether 4 g of paracetamol daily (in divided doses) results in a more rapid recovery from acute LBP than placebo. A secondary aim is to determine if ingesting paracetamol in a time-contingent manner is more effective than paracetamol taken when required (PRN) for recovery from acute LBP.

**Methods/Design:**

The study is a randomised double dummy placebo controlled trial. 1650 care seeking people with significant acute LBP will be recruited. All participants will receive advice to stay active and will be randomised to 1 of 3 treatment groups: time-contingent paracetamol dose regimen (plus placebo PRN paracetamol), PRN paracetamol (plus placebo time-contingent paracetamol) or a double placebo study arm. The primary outcome will be time (days) to recovery from pain recorded in a daily pain diary. Other outcomes will be pain intensity, disability, function, global perceived effect and sleep quality, captured at baseline and at weeks 1, 2, 4 and 12 by an assessor blind to treatment allocation. An economic analysis will be conducted to determine the cost-effectiveness of treatment from the health sector and societal perspectives.

**Discussion:**

The successful completion of the trial will provide the first high quality evidence on the effectiveness of the use of paracetamol, a guideline endorsed treatment for acute LBP.

**Trail registration:**

ACTRN12609000966291.

## Background

Low back pain is common and expensive. The point prevalence is reported to be as high as 33% [1] and 50% of people who have low back pain are expected to seek care [2]. More than AU$1 billion is spent each year on treatments, while indirect costs to Australian society are greater than AU$8 billion per annum [3]. In the United States, the figure is over US$50 billion[4]. Despite the large number of trials investigating treatments for low back pain, few treatments have demonstrated better outcome for patients compared to first line care endorsed in clinical practice guidelines [5].

Clinical practice guidelines aim to provide the practitioner with evidence-based recommendations upon which to base patient management. Such guidelines for acute low back pain have been published in over 11 countries and provide clear agreement on the recommendations for first line care of acute low back pain [6]. According to most guidelines first line care should consist of reassurance on the favourable prognosis of non-specific low back pain, advice to stay active and avoid bed rest, and simple analgesic medication recommended on a time-contingent dose regimen, eg 1 g paracetamol administered 4 times per day. The provision of more complex and expensive interventions is advised if the patient does not respond to first line care.

Despite broad development and publication of clinical practice guidelines for low back pain worldwide, usual care provided to patients appears to be dissimilar to the evidence-based recommendations [7-12]. A common finding is that non-steroidal anti-inflammatory drugs are used in preference to paracetamol for pain relief. While guidelines recommend regular doses of paracetamol (4 g daily in divided doses), another common finding is that when paracetamol is recommended the dose is often sub-therapeutic or it is administered as required (*pro re nata*, PRN), rather than on a time contingent basis [11]. A possible reason for why paracetamol is often not prescribed and/or not used as recommended is that the use of paracetamol for acute low back pain at any dose has no direct evidence supporting its effectiveness [5,13,14]. Guideline recommendations to use paracetamol as first line care thus stem from the relatively low risk of side effects it presents with comparable benefits when compared to other analgesics [6,15].

Hancock et al [16] showed that adding treatments (non steroidal anti-inflammatory drugs or spinal manipulative therapy) to guideline recommended first line care, for patients with acute low back pain, did not improve patient outcomes. Although the study does not provide direct evidence for the efficacy of paracetamol, it found that patients who received 4 g of paracetamol daily (in divided doses) recovered rapidly: approximately 50% of the patients had completely recovered within 2 weeks and 75% within 4 weeks. These rates are faster than previously reported in the literature [17,18] and suggest that an analgesic regimen as simple as 4 g per day of paracetamol may be a highly effective treatment for acute low back pain. However, without direct evidence for the efficacy of paracetamol in low back pain, it may continue to be overlooked as a credible treatment.

The primary aim of this study is to investigate whether taking paracetamol results in faster recovery times when compared to placebo for patients with acute low back pain. Further, because guidelines recommend that adequate, regular doses of paracetamol need to be administered, this study will also investigate if taking paracetamol as a time-contingent dose regimen results in more rapid recovery than taking paracetamol PRN for pain. Secondary aims of this study are to assess the effect of paracetamol on pain, disability, global perceived effect and sleep quality. To help establish the place of paracetamol in managing acute low back pain this study we will also conduct a cost-effectiveness analysis of paracetamol for low back pain and will determine whether specific variables influence a patient's response to treatment.

## Methods/Design

The study will be a three arm randomised double dummy, placebo controlled trial. Ethics approval has been obtained from the University of Sydney Human Research Ethics Committee.

### Study Population

One thousand six hundred and fifty people from the community who consult a general practitioner (GP) with acute non-specific low back pain will be recruited. To be eligible to enter the trial all participants must satisfy the following criteria:

▪ Primary complaint of pain in the area between the 12^th ^rib and buttock crease, with or without leg pain;

▪ Experiencing a new episode of low back pain, preceded by a period of at least one month without low back pain;

▪ Pain of less than 6 weeks duration (in accordance with the Cochrane Collaboration Back Review Group definition for 'acute' pain)[19];

▪ Low back pain severe enough to cause moderate pain (as measured by adaptations of item 7 of the SF-36);

▪ Not have known or suspected serious spinal pathology (eg metastatic, inflammatory or infective diseases of the spine, cauda equina syndrome, spinal fracture);

▪ Not be currently taking recommended regular doses of analgesics, including paracetamol;

▪ Not have had spinal surgery within the preceding 6 months;

▪ Not have serious co-morbidities preventing prescription of paracetamol eg: liver or renal failure;

▪ Not currently taking psychotropic medication and whose health condition is considered to prevent reliable recording of study information;

▪ Not be pregnant or planning to become pregnant during the treatment period.

### Randomisation and Blinding

Eligible patients will be randomised to one of three treatment groups. Randomisation will be performed in the following manner. A researcher not involved in patient recruitment or data collection will create the randomisation schedule by using the random number function in EXCEL. An independent company will then prepare the treatment packs using the randomisation schedule and a researcher not involved with data collection will cross check the intended ingredients of a random sample of the treatment packs to the randomisation schedule to ensure no errors have occurred.

When the GP determines a patient is eligible to enter the trial the patient will be supplied with a pre-randomised, treatment pack containing the study medicines. A researcher will then contact the eligible patient to reconfirm the patient's eligibility and then instruct them to break the seal on the randomised medication box. This contact will occur within 24 hours, where possible, but no longer than one week from the time of recruitment. After the eligibility criteria are confirmed and the seal on the medication box has been broken, the patient will be considered to have been randomised to the trial.

Each treatment pack will contain four coloured medicine boxes (two red and two white). Red boxes are for medicines to be taken on a time-contingent basis and white boxes are for PRN medicines. The active medication differs between each group as follows:

1. Active time-contingent group (Active red pack and placebo white pack);

2. Active PRN group (Placebo red pack and active white pack);

3. Placebo group (Placebo red pack and placebo white pack).

The placebo tablet used in this study is identical to the active tablet but does not contain the active ingredient (paracetamol). All medication packaging is identical with a randomised number printed on the sealed outer box. The GP, participant and researchers involved in data collection will be blind to group allocation. To assess adequate blinding of treatment allocation participants will be asked at Week 12 if they believed they took an active time contingent medicine, an active PRN medicine or two placebo medicines.

### Treatments

Participating GPs will be trained on study methods and procedures individually or in small groups. Specifically, GPs will be trained in administering the study treatment (paracetamol and standard advice as recommended in international evidence based guidelines for acute low back pain [5,6]).

GPs will be provided with a manual outlining the study procedures and worksheets for participant recruitment (participant screening checklist, ineligible patient log, treatment checklist and forms for informed consent).

All participants will be given advice (to avoid bed rest and stay active) and reassurance of a favourable prognosis by their GP [6]. In addition, all participants will be asked to take medications from both the red (time-contingent) and white (PRN) packs. Table 1 shows group medication scheduling. Participants will receive only one active medication and therefore a maximum of 4 g of paracetamol daily as recommended by the Australian Medicines Handbook (2008). Each active time-contingent dose regimen contains 665 mg of extended release paracetamol tablets. The instructions for the time-contingent medications will be to take two tablets three times daily (TID), morning, noon and evening. Therefore, participants in the active time-contingent group will receive 3990 mg of paracetamol per day. Each active PRN tablet will contain 500 mg of immediate release paracetamol. The instructions for the PRN medications will be to take 1-2 tablets as required for pain to a maximum of 8 tablets per 24 hours. Participants in the active PRN group will therefore take up to 4 g of paracetamol per day. The participants in the placebo group will receive two inactive (placebo) tablets, identical to the active medication but containing no paracetamol. All patients will be asked to continue taking the medicines (active or placebo) until they have experienced seven consecutive days of pain rated 0 or 1 out of 10 on a numerical rating scale, or for a maximum of 4 weeks.

**Table 1:** Medication schedule for all treatment arms

Pack	Schedule	Group		
		**Time-contingent** **paracetamol**	**PRN** **paracetamol**	**Placebo**

Red	Morning Noon Night	2 active tablets 2 active tablets 2 active tablets	2 placebo tablets 2 placebo tablets 2 placebo tablets	2 placebo tablets 2 placebo tablets 2 placebo tablets

				

White	As required	1-2 placebo tablets as required to a maximum of 8 per 24 hours	1-2 active tablets as required to a maximum of 8 per 24 hours	1-2 placebo tablets as required to a maximum of 8 per 24 hours

### Co-interventions

Participants will be asked to return to their GP for review 1 week after randomisation and not to receive any other treatments for their back pain. Participants who experience high levels of continuing or worsening pain will be able to return to their GP for an earlier review. Further examination will be provided by the GP and in some instances, a rescue medication (Naproxen 250 mg) can be provided in addition to the study medicine. The criteria for providing a patient with the rescue medication will be based on continuing and worsening pain that is debilitating in nature (in the short term) or continuing high levels of pain that have not improved after 7 days of treatment, despite following the advice and paracetamol regimen. Instructions for the rescue medication will be to take 2 tablets initially and then 1 tablet every 6 to 8 hours as required for pain relief to a maximum of 5 tablets in 24 hours. Participants will be provided with rescue medication for 2 days duration. The study will record any additional treatments patients receive in addition to the advice and paracetamol regimen, when they took them and how often.

### Baseline Assessment

Baseline data will be collected, by a researcher blind to group allocation, in a telephone interview, before the participant starts taking the medications. During the interview, the researcher will reiterate the advice given to the participant by the GP and confirm the participants understanding of the medication instructions. At baseline, data on socio-economic characteristics, general health, previous and current history, and psychological characteristics will be collected (see additional file 1). Measures of the participants' status at baseline include:

▪ numerical pain rating scale (0-10 scale) [20];

▪ condition-specific measure of disability (Roland Morris Disability Questionnaire, 0-24 scale) [20];

▪ patient-generated measure of function (Patient-Specific Functional Scale, 0-10 scale) [20];

▪ global perceived effect (-5 to 5 scale) [20];

▪ quality of life (SF 12) [21];

▪ sleep quality (Pittsburgh Sleep Quality Index item 6) [22];

▪ treatment credibility (Credibility Expectancy Questionnaire) [23].

### Follow-up procedure

A researcher blind to the treatment allocation will contact participants weekly for the four-week treatment period until recovery and, if recovery has not occurred, every two weeks for the remainder of the three-month follow up period or until recovery. The researcher will transcribe the participant's responses, previously recorded in their individual assessment manuals, into a separate manual and into a database.

### Outcomes

The primary outcome will be days to recovery, which is defined in two ways, pain intensity rated as 0 or 1 out of 10 on a 0-10 numerical rating scale (NRS) for seven consecutive days or the first day the participant has a rating of 0 out of 10 on the 0-10 NRS. This will be captured in a daily pain and medication use diary that participants will complete until recovery.

Secondary outcomes are pain, disability, function, global perceived effect and sleep quality. These data will be captured at weeks one, two, four and twelve post randomisation. These outcomes will allow us to evaluate the hypothesised role of regular paracetamol or PRN paracetamol in improving the clinical course of acute low back pain. We will also perform an economic evaluation of paracetamol treatment. For this purpose, data on quality of life, health care and community service utilisation and absenteeism (days of reduced work activity) for the study period will be collected at baseline, 4 weeks and 12 weeks.

### Adherence

Adherence with the study medication treatment will be assessed in three ways; daily self-recorded medication intake, counts of returned tablets following the completion of treatment and a compliance questionnaire (Brief Adherence Rating Scale)[24] administered at Week 4. Medication adherence will be encouraged at week 1 and 2 follow-up interviews. Tablet boxes and blister foils will be labelled with the name of the study and give instructions for scheduling of the medicine. Participants will be asked to return all unused tablets for counting at the end of the treatment period in a reply paid post satchel.

### Data Integrity and analysis

The integrity of trial data will be monitored by regularly scrutinising data files for omissions and errors. All data will be double entered and the source of any inconsistencies will be explored and resolved. For the primary outcome (days to recovery) we will cross check documents transcribed by researchers in follow up interviews with participant diaries returned with the unused medications. If there is discrepancy, the diaries will be used as the source data. Where participant documents are not returned, data transcribed by researchers will be considered source data.

Data will be analysed by a statistician who is blinded to group status. The primary analyses will be by intention-to-treat and we will restrict the number of analyses in order to reduce the possibility of Type I errors. For primary outcomes, a p-value of < 0.05 will be considered statistically significant. For the secondary outcomes a p-value of < 0.01 will be considered significant.

### Primary Statistical Analysis

The difference in survival curves (days to recovery from pain) will be assessed for the three groups using the log-rank statistic. The median days to recovery will be used to express the time to recovery for the three groups. Cox regression will be used to assess the effect of treatment group on hazard ratios after allowing for baseline pain scores, number of previous episodes and duration of episode as covariates.

### Secondary Statistical Analysis

Linear models will be used to assess the effect of treatment group on secondary outcomes at one, two, four and twelve weeks. Specific comparison will be the effect of time contingent paracetamol on secondary outcome measures (pain intensity, disability, function, GPE and sleep quality) compared to PRN paracetamol or placebo.

A cost-utility analysis will be conducted. The primary analysis will be conducted from the health sector's perspective, where costs of healthcare services will be valued at standard rates published by the Australian Government: the Medical Benefits Scheme standard fees for medical services and procedures, the Pharmaceutical Benefits Scheme cost for medications and the Australian Refined Diagnosis Related Groups (AR-DRG) cost weights for hospital services. Private non-medical healthcare services (e.g. physiotherapy) will be valued at standard rates published by the relevant professional body or third party payer.

A secondary analysis will entail a societal perspective in which costs associated with the use of community services and absenteeism will be included. Costs of community services will be based on the self-reported costs of participants. The costs of absenteeism from paid employment will be estimated by the number of days absent from work multiplied by the average wage rate.

Health state utilities required to estimate quality-adjusted life-years (QALY) will be based on measures obtained from the SF-12 and transformed into health state utilities via the SF-6D algorithm[21]. Sensitivity analysis will test uncertainty in key parameters such as the selection of cost weights and statistical variation in quality of life scores.

A secondary analysis to identify predictors of poor outcome in patients receiving guideline based first line treatment (regular paracetamol and advice) will be performed within treatment arm one (time-contingent paracetamol). We will also analyse potential effect modifiers to paracetamol treatment. For this analysis we will investigate interactions between the potential effect modifiers and allocation to time-contingent paracetamol or placebo groups using methods similar to that used by Hancock et al [25]. A prospective validation of a published prognostic rule for rate of recovery from acute low back pain will also be performed [26].

### Sample Size

The sample size was calculated using ACCorD software (version 2). We calculated that a sample size of 550 participants per group will give 80% power to detect a difference of 3 days median time to recovery between the treatment groups and the control group. These calculations are based upon the median days to recovery being 14 in the time-contingent paracetamol group, a rate observed previously [16] and allow for up to 10% treatment non-compliance and a two side alpha of 0.05.

## Discussion

This paper presents the design and rationale for a randomised placebo controlled trial examining the effect of paracetamol on the recovery of patients with acute low back pain. The trial will also determine if paracetamol administered as a time-contingent dose regimen is more effective than paracetamol administered PRN. The primary outcome will be days to recovery. Secondary outcomes will be pain, disability, global perceived effect and sleep quality measured at 1 week, 2 weeks, 4 weeks, and 3 months. We will perform a secondary analysis to determine the cost-effectiveness of treatment and predictors of outcome. The completion of this trial is expected by 2012.

## Competing interests

This investigator initiated study has been awarded funding from the National Health and Medical Research Council, Australia. Subsequent to the award of the NHMRC funding, industry funding was obtained to provide the study medications and supplementary funding for the trial. The investigators maintain full autonomy in the design, conduct and reporting of the study.

## Authors' contributions

CMW, JL, CGM, AJM, MJH, ROD, CWCL and JHM were responsible for the design of the study. CGM, JL, CGM, AJM, CWC and MJH procured funding.

CMW drafted the manuscript and all authors have contributed to the manuscript. All authors have read and approved the final manuscript.

## Pre-publication history

The pre-publication history for this paper can be accessed here:

http://www.biomedcentral.com/1471-2474/11/169/prepub

## Supplementary Material

Additional file 1 **Items measured at baseline assessment**. The file lists items measured at baseline, detailing the wording of the questions asked, the responses and a source of reference. Click here for file

## References

[B1] Walker BF The Prevalence of Low Back Pain: A Systematic Review of the Literature from 1966 to 1998 J Spinal Disord 2000 13 205 217 10.1097/00002517-200006000-00003 10872758

[B2] Walker B Muller R Grant W Low back pain in Australian adults. Health provider utilization and care seeking J Manipulative Physiol Ther 2004 27 327 335 10.1016/j.jmpt.2004.04.006 15195040

[B3] Walker B Muller R Grant W Low back pain in Australian adults: the economic burden Asia Pac J Public Health 2003 15 79 87 15038680 10.1177/101053950301500202

[B4] Deyo RA Low-back pain Sci Am 1998 279 48 53 10.1038/scientificamerican0898-48 9674171

[B5] NHMRC Evidence-Based Management of Acute Musculoskeletal Pain: A Guide for Clinicians 2004 NHMRC. Canberra

[B6] Koes BW van tulder M Ostelo R Burton K Waddell G Clinical Guidelines for the Management of Low Back Pain in Primary Care; An International Comparison Spine Spine 2001 26 2504 2514 10.1097/00007632-200111150-00022 11707719

[B7] Tacci JA Webster BS Hashemi L DC C Clinical practices in the management of new-onset, uncomplicated, low back workers' compensation disability claims J J Occup Environ Med 1999 41 397 404 10.1097/00043764-199905000-00008 10337610

[B8] Schers H Braspenning J Drijver R Wensing M Grol R Low back pain in general practice: reported management and reasons for not adhering to the guidelines in the Netherlands Br J Gen Pract 2000 50 457 640 644 11042916 PMC1313775

[B9] Jackson JL Browning R Impact of national low back pain guidelines on clinical practice South Med J 2005 98 139 43 10.1097/01.SMJ.0000136261.21711.85 15759941

[B10] Gonzalez-Urzelai V Palacio-Elua L L-d-M J Routine primary care management of acute low back pain: adherence to clinical guidelines Eur Spine J 2003 12 589 594 10.1007/s00586-003-0567-2 14605973 PMC3467992

[B11] Williams CM Maher CG Hancock MJ McAuley JH McLachlan AJ Britt H Fahridin S Harrison C Latimer J Low Back Pain and Best Practice Care: A Survey of General Practice Physicians Arch Int Med 2010 170 271 277 10.1001/archinternmed.2009.507 20142573

[B12] Di Iorio D Henley E A D A survey of primary care physician practice patterns and adherence to acute low back problem guidelines Arch Fam Med 2000 9 1015 21 10.1001/archfami.9.10.1015 11115201

[B13] Chou R Huffman LH Medications for acute and chronic low back pain: a review of the evidence for an American Pain Society/American College of Physicians clinical practice guideline Ann Intern Med 2007 147 505 14 17909211 10.7326/0003-4819-147-7-200710020-00008

[B14] Davies RA Maher CG Hancock MJ A systematic review of paracetamol for non-specific low back pain Eur Spine J 2008 17 1423 30 10.1007/s00586-008-0783-x 18797937 PMC2583194

[B15] Roelofs PDDM Deyo RA Koes BW Scholten RJPM van Tulder MW Non-steroidal anti-inflammatory drugs for low back pain Cochrane Database of Systematic Reviews 2008 Issue 1 CD000396 10.1002/14651858.CD000396.pub3 PMC10220428 18253976

[B16] Hancock MJ Maher CG Latimer J McLachlan AJ Cooper CW Day RO Spindler MF McAuley JH Assessment of diclofenac or spinal manipulative therapy, or both, in addition to recommended first-line treatment for acute low back pain: a randomised controlled trial Lancet 2007 370 1638 43 10.1016/S0140-6736(07)61686-9 17993364

[B17] Henschke N Maher CG Refshauge KM Herbert RD Cumming RG Bleasel J York J Das A McAuley JH Prognosis in patients with recent onset low back pain in Australian primary care: inception cohort study BMJ 2008 337 a171 10.1136/bmj.a171 18614473 PMC2483884

[B18] Pengel LHM Herbert RD Maher CG Refshauge KM Acute low back pain: systematic review of its prognosis BMJ 2003 327 7410 323 327 10.1136/bmj.327.7410.323 12907487 PMC169642

[B19] van Tulder M Furlan A Bombardier C Bouter L Updated Method Guidelines for Systematic Reviews in the Cochrane Collaboration Back Review Group Spine 2003 28 1290 1299 10.1097/00007632-200306150-00014 12811274

[B20] Pengel L Maher C Refshauge Responsiveness of pain, disability, and physical impairment outcomes in patients with low back pain Spine 2004 29 879 883 10.1097/00007632-200404150-00011 15082988

[B21] Brazier JE Roberts J The estimation of a preference-based measure of health from the SF-12 Med Care 2004 42 851 59 10.1097/01.mlr.0000135827.18610.0d 15319610

[B22] Buysee DJ Reynolds CF Monk TH Berman SR Kupfer DJ The Pittsburgh Sleep Quality Index: A New Instrument for Psychiatric Practice and Research Psychiatry Res 1989 28 193 192 10.1016/0165-1781(89)90047-4 2748771

[B23] Smeets RJEM Beelen S Goossens MEJB Schouten EGW Knottnerus A Vlaeyen JWS Treatment Expectancy and Credibility Are Associated With the Outcome of Both Physical and Cognitive-behavioral Treatment in Chronic Low Back Pain Clin J Pain 2008 24 4 305 315 10.1097/AJP.0b013e318164aa75 18427229

[B24] Byerly MJ Nakonezny PA Rush JA The Brief Adherence Rating Scale (BARS) validated against electronic monitoring in assessing the antipsychotic medication adherence of outpatients with schizophrenia and schizoaffective disorder Schizophr Res 2008 100 60 9 10.1016/j.schres.2007.12.470 18255269

[B25] Hancock MJ Maher CG Latimer J McLachlan AJ Day RO Davies R Can predictors of responders to NSAIDs be identified in patients with acute low back pain? Clin J Pain 2009 25 659 70 10.1097/AJP.0b013e3181a7ee3a 19920714

[B26] Hancock MJ Maher CG Latimer J Herbert RD McAuley JH Can rate of recovery be predicted in patients with acute low back pain? Development of a clinical prediction rule Eur J Pain 2009 13 1 51 55 10.1016/j.ejpain.2008.03.007 18448369

