# Supplementary material for: PACE - The first placebo controlled trial of paracetamol for acute low back pain: design of a randomised controlled trial
Source: BMC Musculoskelet Disord. 2010 Jul 23;11:169. doi: 10.1186/1471-2474-11-169 (PMC2918542; doi:10.1186/1471-2474-11-169)
Supplement: Additional file 1 — Items measured at baseline assessment. The file lists items measured at baseline, detailing the wording of the questions asked, the responses and a source of reference. [file 1471-2474-11-169-S1.DOCX]

**Appendix 1. Items measured at baseline assessment**

| **Items** | **Question** | **Response options** | **Source** |
| --- | --- | --- | --- |
| Date of birth | Date of birth (dd/mm/yy) | Open |  |
| Gender | Gender? | Male  Female |  |
| Length of symptoms | How many days ago did your back pain start? | Open |  |
| Previous episodes | How many previous episodes of low back pain have you had? An episode is defined as pain lasting more than 24 hours which was preceded by at least 1 month without pain. | Open |  |
| Time to present to care | Date presented to GP | Open |  |
| Pain referral | Does your back pain extend beyond your knee? | Yes  No |  |
| ADL limitation | In relation to the current episode, on how many days did back or leg pain force you to cut down on the things you usually do, for more than half a day? | ___ days | [1] |
| Depression | How much have you been bothered by feeling depressed in the past 7 days? | Scale 0-10  0 - Not at all  10 - Extremely | [2] |
| Risk of persistent pain | In your view, how large is the risk that your current pain may become persistent? | Scale 0-10  0 - No risk  10 - Very large risk | [2] |
| Compensation status | Is your back pain compensable? E.g. workers compensation, 3^rd^ party | Yes  No |  |
| Current medications | Are you currently taking any medications? If yes, please detail: | Yes  No  Detail:  Name  Strength  Dose (tablets/day) |  |
| Socioeconomic status | Postcode | Open |  |
|  | Are you in paid employment? If yes what do you do for a living? | Yes  No  If yes - open response.  Researcher categorizes to:  Manager  Professional  Technician and Trade Worker  Community and Personal Service Worker  Clerical and Administrative Worker  Sales Worker  Machinery Operator and Driver Labourer |  |
|  | What is your gross *weekly* household income (annual in brackets)? | Negative of nil income  $1-$649 ($1-$33,799  $650-$1,699 ($33,800-$88,399)  $1,700-$3,999 ($88,400-$207,999)  $4,000 or more ($208,000 or more) |  |
|  | Health insurance status | None  Private hospital only  Private ancillary only  Private hospital and ancillary  DVA |  |

References:

1. Deyo RA, Battie M, Beurskens AJ, Bombardier C, Croft P, Koes B, Malmivaara A, Roland M, Von Korff M, Waddell G: **Outcome measures for low back pain research. A proposal for standardized use.** *Spine* 1998, **23**(18):2003-2013.

2. Linton SJ, Hallden K: **Can we screen for problematic back pain? A screening questionnaire for predicting outcome in acute and subacute back pain**. *Clin J Pain* 1998, **14**(3):209-215
